# Non-Invasive Detection of Coronary Endothelial Response to Sequential Handgrip Exercise in Coronary Artery Disease Patients and Healthy Adults

**DOI:** 10.1371/journal.pone.0058047

**Published:** 2013-03-11

**Authors:** Allison G. Hays, Matthias Stuber, Glenn A. Hirsch, Jing Yu, Michael Schär, Robert G. Weiss, Gary Gerstenblith, Sebastian Kelle

**Affiliations:** 1 Department of Medicine, Division of Cardiology, Johns Hopkins University, Baltimore, Maryland, United States of America; 2 Department of Radiology, Division of Magnetic Resonance Research, Johns Hopkins University, Baltimore, Maryland, United States of America; 3 Department of Biomedical Engineering, Johns Hopkins University, Baltimore, Maryland, United States of America; 4 Department of Radiology, Centre Hospitalier Universitaire Vaudois, Center for Biomedical Imaging (CIBM) and University of Lausanne, Lausanne, Switzerland; 5 Department of Medicine, Division of Cardiology, University of Louisville, Louisville, Kentucky, United States of America; 6 Philips Healthcare, Cleveland, Ohio, United States of America; 7 Department of Medicine, Division of Cardiology, Deutsches Herzzentrum Berlin, Berlin, Germany; University Medical Center Utrecht, The Netherlands

## Abstract

**Objectives:**

Our objective is to test the hypothesis that coronary endothelial function (CorEndoFx) does not change with repeated isometric handgrip (IHG) stress in CAD patients or healthy subjects.

**Background:**

Coronary responses to endothelial-dependent stressors are important measures of vascular risk that can change in response to environmental stimuli or pharmacologic interventions. The evaluation of the effect of an acute intervention on endothelial response is only valid if the measurement does not change significantly in the short term under normal conditions. Using 3.0 Tesla (T) MRI, we non-invasively compared two coronary artery endothelial function measurements separated by a ten minute interval in healthy subjects and patients with coronary artery disease (CAD).

**Methods:**

Twenty healthy adult subjects and 12 CAD patients were studied on a commercial 3.0 T whole-body MR imaging system. Coronary cross-sectional area (CSA), peak diastolic coronary flow velocity (PDFV) and blood-flow were quantified before and during continuous IHG stress, an endothelial-dependent stressor. The IHG exercise with imaging was repeated after a 10 minute recovery period.

**Results:**

In healthy adults, coronary artery CSA changes and blood-flow increases did not differ between the first and second stresses (mean % change ±SEM, first vs. second stress CSA: 14.8%±3.3% vs. 17.8%±3.6%, p = 0.24; PDFV: 27.5%±4.9% vs. 24.2%±4.5%, p = 0.54; blood-flow: 44.3%±8.3 vs. 44.8%±8.1, p = 0.84). The coronary vasoreactive responses in the CAD patients also did not differ between the first and second stresses (mean % change ±SEM, first stress vs. second stress: CSA: −6.4%±2.0% vs. −5.0%±2.4%, p = 0.22; PDFV: −4.0%±4.6% vs. −4.2%±5.3%, p = 0.83; blood-flow: −9.7%±5.1% vs. −8.7%±6.3%, p = 0.38).

**Conclusion:**

MRI measures of CorEndoFx are unchanged during repeated isometric handgrip exercise tests in CAD patients and healthy adults. These findings demonstrate the repeatability of noninvasive 3T MRI assessment of CorEndoFx and support its use in future studies designed to determine the effects of acute interventions on coronary vasoreactivity.

## Introduction

Coronary responses to endothelial-dependent interventions are important measures of vascular risk, predicting early and late cardiovascular events [1], [2], [3], [4], [5], [6]. However, the measurement of coronary endothelial function previously required invasive coronary angiography to quantify the vasodilatory and flow responses to endothelial-dependent stressors. This invasive requirement limited clinical and research investigation of coronary endothelial function, particularly in healthy and in low risk subjects, as well as the performance of repeated studies over time. Recently developed MRI methods, however, are now capable of quantifying coronary endothelial vasoreactivity non-invasively with excellent intra- and inter-observer reproducibility [7], [8], [9] using isometric handgrip exercise as the endothelial-dependent stressor. This technique enables safe, repeated studies of coronary endothelial function in an expanded population. However, in order for this MRI method to accurately quantify the coronary endothelial response to interventions, additional studies are needed.

Sequential studies allowing paired comparisons of coronary artery area and blood flow responses to endothelial-dependent stresses before and following an acute intervention are used to assess the endothelial response to that intervention. Because coronary endothelial function may change over a short time period (minutes to hours) in response to environmental stimuli or intervention [10], [11], [12], this paradigm is only valid if the second response does not differ from the first in the absence of an intervention under normal conditions. Prior studies show evidence of a positive “training effect” for endothelial function in coronary artery disease (CAD) patients, wherein an initial abnormal response is followed by improvement after weeks of exercise training [13], [14], [15], [16]. In contrast, the endothelial response in healthy subjects, which is normal initially, is unchanged on the second exam. In those studies, the second test was performed after weeks, a relatively long time period. Addressing the question of any change in repeated studies over a shorter time frame would be important in laying the framework for future studies intended to assess short term effects of therapeutic interventions on coronary endothelial function. We therefore sought to test the hypothesis that coronary endothelial vasoreactivity does not differ between first and second isometric handgrip (IHG) exercise separated by a short ten-minute period in CAD patients and in healthy adults.

## Methods

### Patients

The protocol was approved by the Institutional Review Board at The Johns Hopkins School of Medicine and all participants provided written informed consent. No subject had a contraindication to MRI. Healthy subjects were those under age 50 years without a known history of CAD and traditional CAD risk factors, and for those over age 50 years, an Agatston coronary artery calcium score <10 [17]. CAD subjects were outpatients with coronary artery disease (>30% stenosis) on coronary x-ray angiography within the 12 months preceding study enrollment.

### Study Protocol

**Figure 1:**
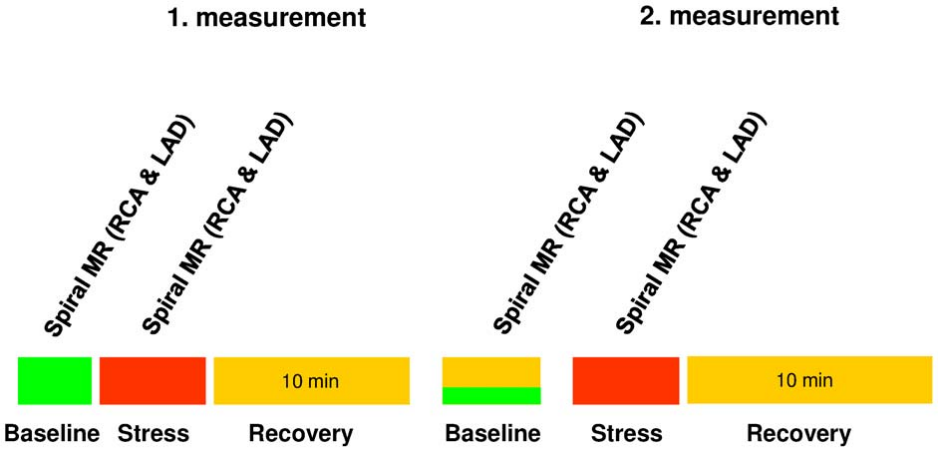
Diagram illustrating MRI study flow with measured parameters. Hemodynamic parameters have been measured at all time-points (blood pressure and heart rate).

**Figure 2:**
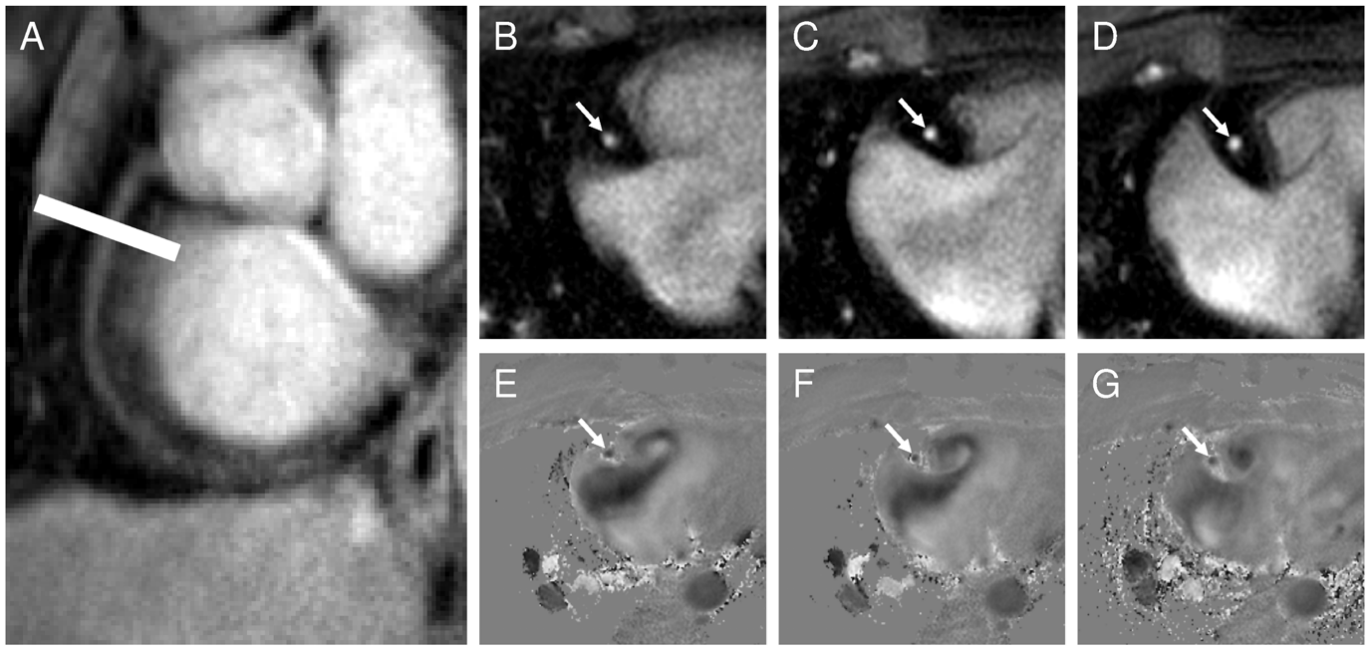
Typical anatomical and flow-velocity encoded coronary images using magnetic resonance imaging at rest and with sequential isometric handgrip stresses in a healthy subject. In image (**A**), a scout scan obtained parallel to the RCA is shown together with the location for cross-sectional imaging (white line). (**B**) shows (white arrow) the region (corresponding to the cross-sectional location from A) that was selected for analysis at rest (**B**), during the first handgrip stress (**C**) and second handgrip stress (**D**). The white arrow in E shows a cross-section of the RCA that was selected for analysis of coronary flow velocity measures in the healthy volunteer. The signal intensity is proportional to flow velocity with a black signal indicating high velocity down through the imaging plane. In the view of the RCA (white arrow) at baseline (**E**) and during the first handgrip stress (**F**) and second handgrip stress (**G**) the change in luminal coronary signal intensity (increased blackness) indicates a proportional change in through-plane coronary flow velocity.

MRI was performed in the morning in the fasting state before administration of any prescribed vasoactive medications. A diagram illustrating MRI study flow with measured parameters is shown in **Figure 1**. Images were taken perpendicular to a proximal, linear segment of the coronary artery best identified on scout images. To ensure slice orientation perpendicular to the coronary artery, double oblique scout scanning was performed as previously reported [18]. The imaging plane for the endothelial function measurements was localized in a proximal or mid arterial segment that was straight over a distance of approximately 20 mm (**Figure 2** **, A**). All acquisitions were performed during a pre-specified period of least cardiac motion [19]. In some cases, two coronary arteries per participant were imaged when both arteries displayed equivalent image quality, and the results for each were quantified and reported.

Baseline imaging at rest for cross-sectional coronary artery area measurements [20] (**Figure 2** **, B**) was followed by coronary flow velocity-encoded MRI [21]. Coronary artery cross-sectional area and blood flow were quantified before and during two sequential isometric handgrip (IHG) stresses and immediately prior to the second IHG exercise during the recovery period (“pre exercise 2”). Each subject performed sustained isometric handgrip exercise using an MRI-compatible dynamometer (Stoelting, Wood Dale, IL, USA) for four minutes at 30% of their maximum grip strength [22] while being supervised by a research nurse. Heart rate and blood pressure were measured throughout using a non-invasive and MRI-compatible ECG and calf blood pressure monitor (Invivo, Precess, Orlando, FL, USA). The rate pressure product (RPP) was calculated as systolic blood pressure x heart rate. The second IHG study was performed 10 minutes after the completion of the first IHG study.

### MRI

A commercial human 3.0 Tesla (T) whole-body MR scanner (Achieva, Philips, Best, NL) with a 6-element cardiac coil for signal reception was used. Cross-sectional anatomical [20] and flow velocity encoded spiral MRI [21] were performed using single breath-hold cine sequences [23]. MRI parameters for anatomical imaging were: echo time (TE) = 1.5 ms, radio frequency (RF) excitation angle = 20°, breath-hold duration∼14–24 sec, acquisition window = 10 ms, repetition time (TR) = 14 ms, 21 spiral interleaves/cine frame, and spatial resolution = 0.89×0.89×8.0 mm^3^. MRI parameters for the flow measurements were: TE = 3.5 ms, RF excitation angle = 20°, breath-hold duration∼20 seconds, acquisition window = 27 ms, TR = 34 ms, 11 spiral interleaves/cine frame, spatial resolution = 0.8×0.8×8 mm^3^, and velocity encoding = 35 cm per second. The total duration of the MRI exam was ∼ 60 minutes.

### Image Analysis

Images were analyzed for cross-sectional area changes using a semi-automated software tool (Cine version 3.15.17, General Electric, Milwaukee, WI, USA). A circular region-of-interest was manually traced around the coronary artery in diastole during a period of least coronary motion. The computer algorithm employed an automated full width half maximum algorithm for the cross-sectional coronary area measurements.

For flow measurements, images were analyzed using commercially available software (FLOW Version 3.0, Medis, NL). Peak diastolic coronary flow velocity was used for the velocity measurements and coronary artery blood-flow was calculated (and converted to the units mL/minute) using the adapted equation: coronary artery cross-sectional area x coronary artery peak diastolic velocity x 0.3 [24].

### Statistical Analysis

Statistical analysis was performed using SPSS 18.0 for Windows (SPSS Inc). Data are expressed as mean ± standard error. Proportions were compared using chi-square tests. Paired Student’s t-tests were used to compare stress coronary artery cross-sectional area, diastolic coronary flow velocity and blood-flow measurements to the initial baseline measurements obtained prior to stress, and to compare changes in all three parameters between the first and second stress. Student’s unpaired t-tests were used to compare the changes from rest to stress in coronary cross-sectional area, peak diastolic coronary flow velocity, and blood-flow measurements between the healthy and CAD subjects. The data were tested for normality using the Shapiro-Wilk test and the results indicated that parametric testing was appropriate. The Bland-Altman method was used to assess interobserver and intraobserver agreement for area, peak diastolic velocity and coronary blood-flow measurements with p-values derived from Pitman’s test of differences. Statistical significance was defined as a two-tailed p-value <0.05.

## Results

**Figure 3:**
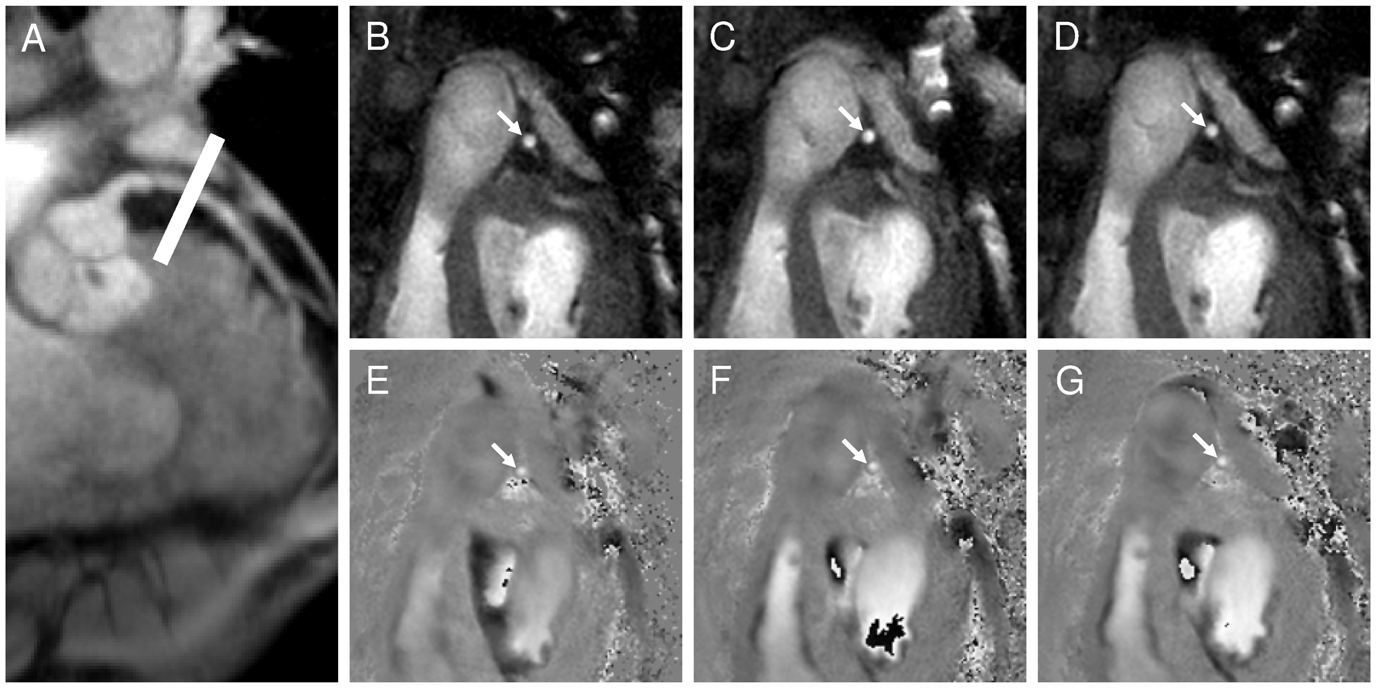
Typical anatomical and flow-velocity encoded coronary images using magnetic resonance imaging at rest and during sequential isometric handgrip stress in a CAD patient. A scout scan obtained parallel to the left anterior descending (LAD) artery (**A**) is shown together with the location for cross-sectional imaging (white line). The corresponding cross-section of the LAD is shown at rest (**B**) and during the first (**C**), and second handgrip stress (**D**, white arrows) and indicates no significant change in coronary cross sectional area during each stress. The white arrow in **E** shows a velocity-encoded image of the same LAD cross section at rest, during the first handgrip (**F**) and second handgrip stress (**G**). In this case, because the direction of blood flow is being analyzed in the LAD, the change in luminal coronary signal intensity (degree of “whiteness”) indicates a proportional change in through-plane coronary flow velocity.

Seventeen of twenty healthy subjects (85%) and eleven of twelve CAD patients (92%) completed the study with adequate image quality. Three healthy subjects were excluded due to broken coil (N = 1), non-diagnostic image quality because of bulk movement (N = 1) and incomplete study due to shoulder pain (N = 1). One CAD patient was excluded because of non-diagnostic image quality. Thirty coronary artery segments in 17 healthy subjects and 15 coronary artery segments in 11 CAD patients were evaluable for analysis (**Figures 2** and **3** **)**. Baseline characteristics of the study population are presented in **Table 1**.

**Table 1:** Characteristics of the Subjects.

Characteristics	Healthy Subjects N = 17	CAD Patients N = 11	P value
Age (yr), Mean ±SD	31±10	57±6	<0.001
Gender (male)	8 (47)	8 (73)	NS
Previous MI	0	5 (45)	0.016
PCI/stent	0	7 (64)	0.002
CABG	0	1 (9)	0.34
CAD risk factors*			
History of smoking	0	3 (27)	0.08
Dyslipidemia	0	9 (82)	<0.001
Diabetes mellitus	0	0	NS
Hypertension	0	8 (73)	<0.001
Family history of early CAD	0	3 (27)	0.08
Vessel/s studied			
RCA	15	7	0.84
LAD	15	5	0.30
LCX	0	3	0.08
Total vessels studied	30	15	

Abbreviations; SD = standard deviation, CAD = coronary artery disease, PCI = percutaneous coronary intervention, CABG = coronary artery bypass graft surgery, MI = myocardial infarction, RCA = right coronary artery, LAD = left anterior descending coronary artery, LCX = left circumflex coronary artery.

* CAD risk factors excluding age and gender.

### Hemodynamic Effect of Isometric Handgrip (IHG) Stress

**Figure 4:**
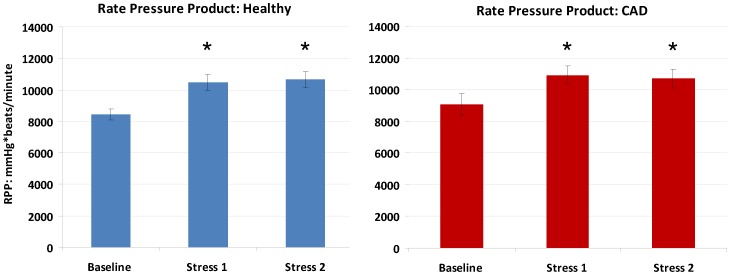
Rate pressure product (RPP, systolic blood pressure X heart rate) is shown at baseline and during isometric handgrip stress (first and second) in both healthy subjects (blue bars) and coronary artery disease patients (red bars). * signifies p<0.05 compared to baseline RPP. Error bars indicate standard error of the mean.

IHG exercise caused a significant hemodynamic effect in both groups. In healthy subjects, the baseline rate pressure product (RPP, heart rate x systolic blood pressure) of 8437±346 mmHg*beats/minute increased to 10,471±515 mmHg*beats/minute with the first stress (p<0.0001 vs. baseline). RPP increased similarly during the second stress in healthy subjects (Figure 4). In CAD patients, the baseline rate pressure product RPP of 9087±689 mmHg*beats/minute increased to 10,917±548 mmHg*beats/minute with the first stress (p<0.0001 vs. baseline). It also increased comparably during the second stress. For both the healthy subjects and CAD patients, the pre-exercise 2 RPP (taken immediately prior to the 2^nd^ IHG stress at the end of the 10 minute recovery period) was not significantly different from the original baseline value (healthy baseline vs. pre-exercise 2 RPP: 8437±346 mmHg*beats/minute vs. 8339±317, p = 0.61; CAD: 9087±689 mmHg*beats/minute vs. 9385±639, p = 0.09. The change in RPP with stress did not significantly differ between stress 1 and stress 2 for either group (healthy, p = 0.66; CAD, p = 0.76) and between CAD and healthy subjects (stress 1 RPP healthy vs. stress 1 CAD, p = 0.32).

### Coronary Vasodilatation

**Figure 5:**
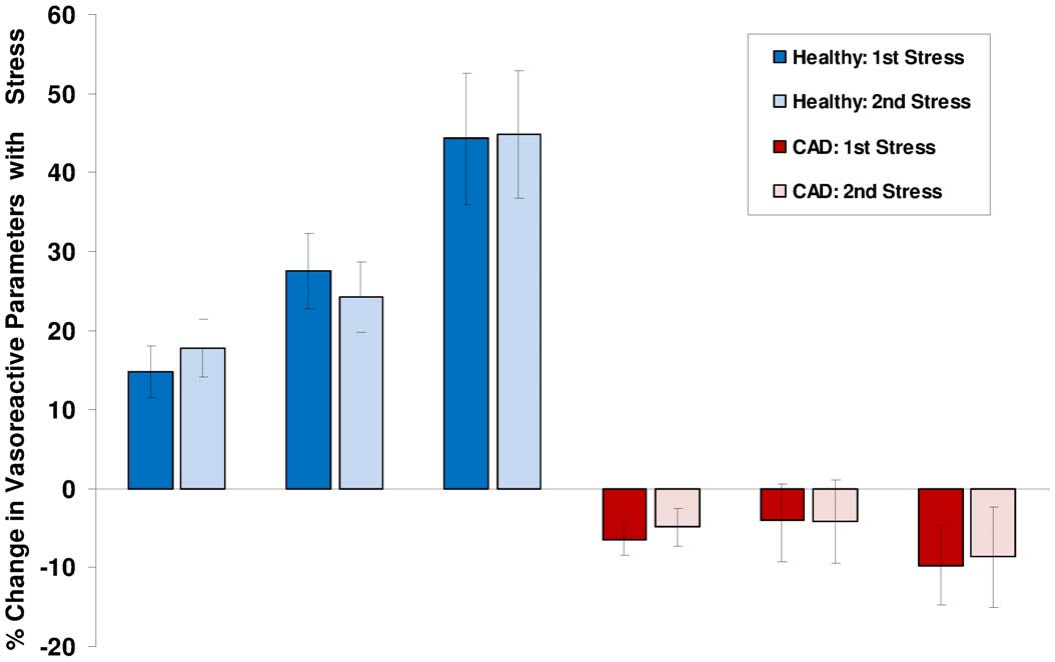
Percent change in coronary endothelial vasoreactive parameters (area, velocity and flow) is shown during first and second isometric handgrip stress for both healthy subjects (blue bars) and CAD patients (red bars). Error bars indicate standard error of the mean. In the healthy group, a normal coronary endothelial response is seen with an increase in coronary artery area, velocity and flow with stress, and no significant difference between stress 1 and stress 2 response. In the CAD group, there is an abnormal coronary endothelial response with no increase or decrease in the same three parameters with stress, and no significant difference in response between stress 1 and 2.

In the healthy group, coronary arteries dilated significantly during the first IHG stress (baseline cross-sectional area: 10.1±0.5 vs. first stress: 11.6±0.7 mm^2^, p<0.0001) and second stress: 11.9±0.7 mm^2^, p<0.0001). There was no significant difference in % cross-sectional area (CSA) change with IHG between the first and second stress (% increase in mean CSA with stress 1∶14.8% ±3.3% vs. stress 2∶17.8% ±3.6%, p = 0.24). In contrast to the increase in CSA in the healthy group, CSA decreased with the first and second stresses in the CAD group (baseline area: 14.0±1.1 vs. stress area 1∶13.1±1.0 mm^2^, p = 0.005, n = 15), and second stress: 13.3±1.0 mm,^2^ p = 0.06), although again the percent change in mean CSA during the two stresses did not differ from one another (−6.4% ±2.0% vs. −5.0% ±2.4% for the first and second studies respectively, p = 0.22). In the healthy group, the coronary artery area measured just before the second exercise period was similar to that measured at baseline (baseline cross-sectional area: 10.1±0.5 mm^2^ vs. pre-exercise 2∶10.3±0.6 mm^2^, p = 0.51), while in the CAD group, the coronary artery area was lower before the second exercise period as compared to baseline (baseline cross-sectional area: 14.0±1.1 mm^2^ vs. pre-exercise 2∶13.1±1.0 mm^2^, p = 0.01). Importantly, there was a significantly different response between healthy subjects and CAD patients in terms of direction and magnitude of coronary vasoreactivity to IHG stress (healthy CSA change (stress 1): 14.8% ±3.3% vs. CAD area change (stress1): −6.4% ±2.0%, p<0.0001). The relative stress-induced area changes in both groups are shown in **Figure 5**.

### Coronary Flow Velocity and Blood-flow Measures

Peak diastolic coronary flow velocity increased in healthy subjects during the first and second stresses (20.7±0.9 cm/s baseline vs. 26.4±1.3 cm/s and 25.7±1.1 cm/s, for the first and second studies respectively, p<0.0001 vs. baseline) with no significant difference in the percent velocity change between the two tests (p = 0.54). There was no significant change in peak diastolic flow velocity with stress for CAD subjects (baseline vs. stress1∶20.0±1.4 cm/s vs. 19.2±1.5 cm/s, p = 0.42; and vs. stress2∶19.2±1.4 cm/s, p = 0.53). In the healthy and CAD groups, there was no significant difference in velocity values between the baseline and pre-exercise 2 measurements (healthy: baseline velocity: 20.7±0.9 cm/s vs. pre-exercise 2∶20.1±0.7 cm/s, p = 0.28; CAD: baseline velocity: 20.0±1.4 cm/s vs. pre-exercise 2∶18.4±1.0 cm/s, p = 0.09).

Coronary blood flow increased significantly with IHG stress in healthy subjects and decreased in CAD patients (healthy flow change (stress 1): 44.3% ±8.3% vs. CAD flow change (stress1): −9.7% ±5.1%, p<0.0001). In healthy subjects, coronary blood-flow increased significantly with isometric handgrip during both stress periods (baseline: 63.2±4.6 ml/minute vs. stress 1∶91.2±6.2 ml/minute, p<0.0001, and vs. stress 2∶91.5±5.9 ml/minute, p<0.0001). In CAD patients, blood-flow did not increase, but decreased slightly with the first and second stresses, although not significantly (baseline: 83.9±9.7 ml/minute vs. stress 1∶75.8±8.0 ml/minute, p = 0.13, and vs. stress 2∶76.6±7.0 ml/minute, p = 0.40). In the healthy group, the coronary flow measured pre-exercise 2 was similar to the baseline value (baseline flow: 63.2±4.6 ml/minute vs. pre-exercise 2 flow: 63.1±5.5 ml/minute, p = 0.93). In the CAD group, the pre-exercise 2 coronary flow did not return to the original baseline value (baseline flow: 83.9±9.7 ml/minute vs. pre-exercise 2 flow: 69.6±5.3 ml/minute, p = 0.03). Relative to baseline coronary blood-flow, changes with stress were not significantly different between stress 1 and stress 2 in either the healthy subjects (stress 1 flow change: 44.3% ±8.3% vs. stress 2∶44.8% ±8.1%, p = 0.84) or the CAD patients (−9.7% ±5.1% vs. stress 2: −8.7% ±6.3%, p = 0.38). Relative changes in velocity and flow for both groups are shown in **Figure 5**.

### Reproducibility

**Figure 6:**
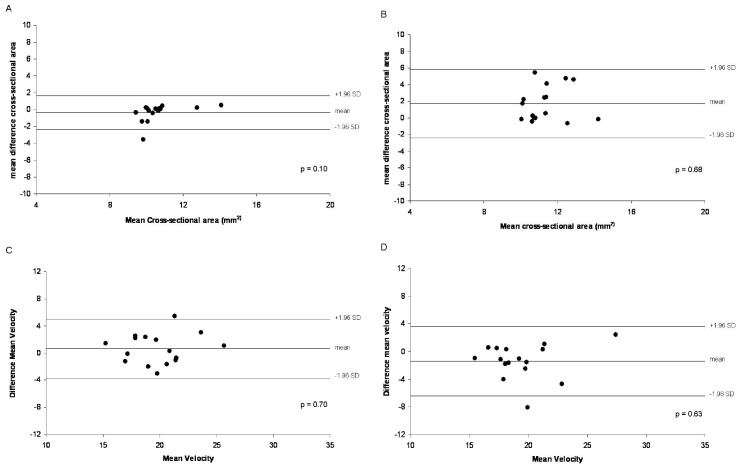
Bland-Altman plots for intra-observer and inter-observer variability. Bland-Altman plots for intra-observer variability (A and C) and inter-observer variability (B and D) of coronary artery cross-sectional area (A and B) and peak diastolic flow velocity (C and D) measurements in CAD patients and healthy subjects. Solid lines above and below the mean represent ±2 standard deviations and the mean differences are shown. P-values are derived from Pitman’s test of differences.

The intra-observer results for area and velocity measurements showed no significant differences (p = 0.10 and p = 0.70 respectively). Similarly, the inter-observer variability for the area and velocity measurements did not show significant differences (p = 0.68 and p = 0.63 respectively) similar to that previously reported [7], [8]. Bland-Altman plots are shown in **Figure 6** **(A–D)**.

## Discussion

3T MRI was performed at rest and during sequential isometric handgrip exercise, an established endothelial-dependent stressor. IHG exercise caused significant hemodynamic effects in healthy and CAD subjects during both stress periods. The coronary endothelial response to stress in the healthy group, as expected, was marked by vasodilation and increased flow. The responses during the first and second stress periods did not differ. In the CAD group, the coronary endothelial responses during the first and second stresses were abnormal with a lack of vasodilation and decreased flow, and these did not differ from the first stress to the second. Therefore, when compared to the unperturbed state (baseline 1), the coronary vasoactive responses to IHG exercise are similar between two successive exercise sessions for both healthy subjects and patients with CAD. However with this protocol where the second IHG exercise commenced 10 minutes after the first, the second pre-exercise coronary indices had not returned to baseline values (those prior to first IHG) in CAD patients, although they did in healthy volunteers. In future studies which may investigate the role of an intervention, it is critical to compare the two IHG responses to the true baseline, unperturbed state. A longer recovery period between the two successive stress intervals could also be investigated in future studies.

The values for coronary endothelial function reported here are similar to those previously reported using MRI [7], [25], [26], [27] and invasive techniques [2], [28], [29], [30], [31] in separate studies, although the endothelial-dependent stressors differed among studies. Although we previously reported excellent reproducibility of the MRI technique in subjects during separate scanning sessions on the same day [7], the immediate effects of repeated IHG on subsequent coronary endothelial response in patients and healthy subjects were not previously studied non-invasively.

In animal studies of coronary arteries, there is evidence that short term exercise training enhances nitric oxide (NO)-mediated coronary dilation [32], and increases endothelial NO synthase activity [33], [34]. Prior studies in CAD patients demonstrated *improved* endothelial dependent responses (ie. to acetylcholine) after exercise training [13], [16], [35], [36]. However, the duration of exercise training was at least weeks before the subsequent endothelial response was studied. In contrast to the CAD patient studies, NO-mediated vasodilator function was not changed in healthy humans following short term forearm muscle training [14], [37]. Thus, although the coronary endothelial responses following weeks of exercise training improve in CAD patients and do not change in healthy subjects, the responses in both groups do not change in our protocol between the two study periods, likely because of the much shorter duration between assessments (minutes) and the lack of an intervention. Therefore, the observation that endothelial function is not significantly changed with sequential IHG stress in healthy subjects and CAD patients suggests that there is no significant “training” effect in the two populations within the parameters of our study, i.e. within minutes.

Thus, the non-invasive MRI technique described here is particularly suitable for evaluating asymptomatic populations and for performing repeated studies in low risk individuals. Although PET can be used to assess coronary blood flow in response to endothelial stressors [38], [39], it is unable to measure epicardial coronary artery area changes with stress while the exposure to ionizing radiation limits repeated studies and its use in low risk populations. Cardiac CT can measure changes in coronary artery area, but not coronary flow. Cardiac CT also exposes subjects to ionizing radiation and contrast agents. Lastly, in our MRI study, the use of an MRI contrast agent (gadolinium) was not necessary, offering the ability to safely study patients with renal dysfunction.

### Limitations

One limitation to this study is that we did not compare MRI-derived measures of coronary vasoreactivity with those obtained using invasive methods such as coronary angiography or Doppler guidewire. As many of the subjects were healthy, an invasive coronary test was not clinically indicated and could not be justified. Moreover, MRI measures of coronary area [40], [41] and blood-flow velocity [42], [43] were validated in prior studies, and our results in terms of both direction and magnitude of the coronary responses are similar to those reported using invasive techniques [2], [29], [30], [31]. Another limitation to this study is the relatively small sample size, particularly in the CAD group. Although only one third of the coronary arteries studied belonged to the CAD group, we observed significant differences in the endothelial-dependent responses between healthy and CAD subjects and characterized the response to sequential stress in both groups in a single scanning session. Lastly, the two groups were not age-matched but that does not prevent assessment of reproducibility in a wide age range of subjects.

### Conclusion

In summary, we report that coronary endothelial function measured non-invasively using MRI does not change with repeated isometric handgrip exercise over the short term in both healthy subjects and those with CAD when compared to the baseline unperturbed state. This ability to non-invasively characterize the coronary endothelial responses to repeated IHG exercise coupled with the reproducibility of the results and the short time required for the MRI protocol may facilitate the design of future studies targeting coronary endothelial responses to acute interventions and contribute to the non-invasive characterization of factors that affect vascular function.
